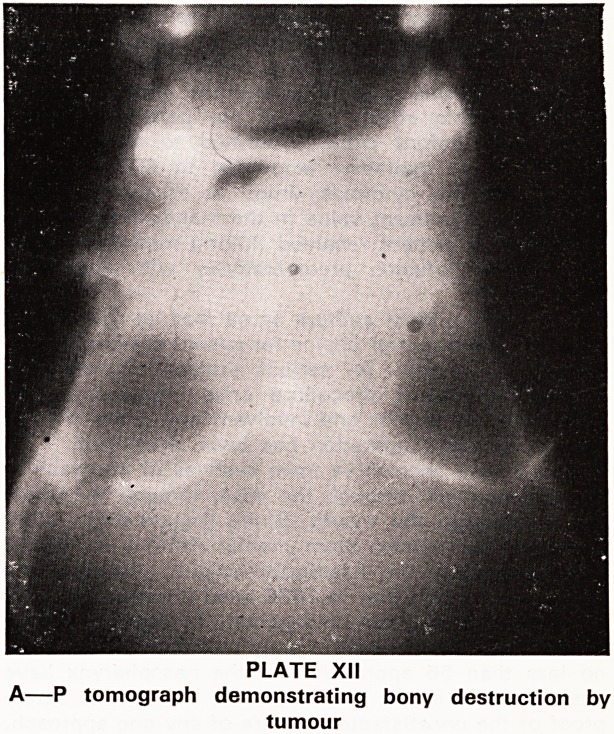# The Management of Nasopharyngeal Angiofibroma
*A Paper read to the Association of Head and Neck Oncologists of Great Britain at their Summer Meeting in Bristol, July 1972.


**Published:** 1974-04

**Authors:** R. Kenneth Roddie

**Affiliations:** Consultant E.N.T. Surgeon, Bristol Clinical Area (Southmead Hospital, Bristol)


### The Management of Nasopharyngeal Angiofibroma*

By
R. KENNETH RODDIE, M.B., F.R.C.S.
Consultant E.N.T. Surgeon, Bristol Clinical Area
(Southmead Hospital, Bristol)

Nasopharyngeal angiofibroma can justifiably be classified as a rare tumour, even though many cases have been reported in the literature. Individual experiences with affected patients are very few, and it is perhaps because of this and the somewhat enigmatic nature of the tumour itself, that the evolution of its management has led to some confusion as to the specific approach to its treatment. Surgical access to the nasopharynx is difficult as attested to by no less than 55 approaches reported in the literature. I have not devised a 56th approach and my personal experience with these tumours is limited.

The purpose of this short paper is to consider the natural history of the tumour, summarize the various therapeutic measures used in its management, and report four patients treated by my colleagues and myself in Bristol during the past ten years.

**Incidence**

The incidence of nasopharyngeal angiofibromata varies in different countries from 1:6,000 to 1:16,000 new clinic visits. As with other neoplasms of the nasopharynx it is more common in Egypt and South-east Asia. There have been few of these tumours recorded in the Negro race. It has long been associated with a predilection for pubescent males, and our four patients were all males in the juvenile age group, their ages ranging from ten to sixteen years. However, various reports in the literature, showing a small percentage incidence in adults, would seem to indicate that this tumour can persist into adult life or appear *de novo* in older patients. If the diagnosis of angiofibroma is made in a female, and Shaheen (1930) had 8% in his series, sex chromosome studies are indicated.

**Pathogenesis**

These tumours do not appear to be true neoplasms. They are locally malignant but never metastasize.

Four theories of origin have been postulated:

(1) *Verneuil (1878, quoted by Bensch)* suggested that the nasopharyngeal angiofibroma originates from the perichondrium of the cartilage joining the basi-occipital bone to the sphenoid. During the second decade of life the cartilage undergoes ossification destroying the site of origin of the tumour. This supports the idea of spontaneous regression, but not of sexual selectivity.

(2) *Brunner (1942)* considered that the tumour originates from the basilar fascia or pharyngeal aponeurosis of the superior constrictor muscle that covers the posterior wall and roof of the nasopharynx.

(3) *Willis (1953)* considered the angiofibroma as a form of immune response, that it developed from an inflammatory allergic state, and was not a true tumour.

(4) *Osborn (1959) and Schiff (1959)* emphasized the basic vascular nature of this tumour. They suggest that the angiofibroma is 'pituitary bound', and is a sexually stimulated cavernous erectile tissue probably of the inferior turbinate type and arises from the nasopharyngeal periosteum. It is in some way associated with the hormonal changes of puberty. None of our patients would appear to substantiate this possible relationship to endocrine imbalance, and hyposexual development was not apparent. However it is generally accepted that hormonal assays in the younger age groups often give inconclusive results.

**Histology**

Histologically the main components of these tumours are numerous arterial and venous channels of large and small calibre, some of which are deficiently developed (Plates VII and VIII). Other thin walled vessels which form large lacunae or smaller channels of capillary size are also present. The second component consists of a large amount of stroma rich in collagen and containing fibroblasts. The profuse bleeding of these tumours may be explained by the absence of elastic fibres, and by the absence or paucity of smooth muscle in the vessel walls. The tumour is not fed by a single large vessel which can be ligated. Its major blood supply is usually from the maxillary and ascending pharyngeal arteries. Biopsy may precipitate severe bleeding, but, after total removal, the bleeding is no greater than that from any large raw surface. Older patients show an increase in fibrous elements, and it has been suggested that the angiofibroma undergoes self-destruction as the angiomatous elements become fibrous, thus causing thrombosis and inflammatory changes in the vessel walls with hyaline degeneration.

* *A Paper read to the Association of Head and Neck Oncologists of Great Britain at their Summer Meeting in Bristol, July 1972.*

PLATE VII
Irregular thin walled vessels forming large lacunae and small channels of capillary size

PLATE VIII
Anomalous vessel of endothelial thickness with absence of smooth muscle in its wall

PLATE IX
Angiofibroma showing multiple protuberant lobulations

PLATE X
Angiofibroma protruding from anterior nares

### Clinical Behaviour

The clinical behaviour mainly depends on the site of origin, rate and direction of growth. If it remains localized in the nasopharynx there may be little in the way of symptoms. In about 25% there is local extension of the tumour. After the age of 20 years the majority either stabilize or regress. It usually originates in the vascular stroma of the connective tissue elements at the base of the sphenoid, as occurred in three of our patients. Occasionally it may arise within similar tissues overlying the pterygoid processes. This was the site of origin in the fourth patient.

There would appear to be two distinct types of expansion of the tumour:

(1) *The primary central growth pattern* consisting of a gradually enlarging smooth tumour. It is spheroid, firm and rubbery in consistency, with a large sessile base. Different islands in the primary tumour grow at different rates and result in multiple protuberant lobulations. The formation of these lobules is somewhat predetermined by the anatomy of the nasopharynx (Plate IX). As it expands it causes erosion by pressure necrosis or direct infiltration. It has the capacity to invade muscle and fascia and to destroy bone. There is no true capsule.

(2) *The second type of growth pattern* consists of long curved elastic extensions into the crevices and recesses of the nasopharynx. Conley (1968) refers to these as *lumbricoid extensions* and they imitate the extensions of neurofibromas of the head and neck region.

*Forward spread* in the ethmoid region may cause widening of the bridge of the nose with separation and protrusion of one or both eyes, ultimately giving rise to the 'frog face' deformity. In one of our patients the tumour protruded from the anterior nares (Plate X).

*Lateral spread* may obstruct the eustachian tube, erode the antral wall and cause swelling of the cheek. It may also invade the pterygoid plate, orbit, sphenoid or cranial cavity, but *not* the dura mater where a plane of cleavage exists. Cranial nerve palsies, especially the 3rd, 4th and 6th can occur. Occasionally it may involve the infratemporal region or present in the posterior portion of the superior gingivobuccal sulcus.

It is this varied character of the expansion of the angiofibroma in the region of the nasopharynx and nasal cavity which adds to the complexity of its management and, in many instances, the lack of success in surgical control.

### Symptoms

The presenting symptoms are due to the mass of tissue, the spread of the tumour and the vascularity. All four patients complained of nasal obstruction, unilateral in three, bilateral in one. Recurrent epistaxis occurred in three, one had retro-orbital pain, two had a conductive deafness associated with eustachian tube obstruction. In two there was rhinolalia clausa due to depression of the soft palate, secondary infection of the paranasal sinuses was present in three. The duration of symptoms varied from 8 to 18 months.

### Diagnosis

Diagnosis is aided by:—

(1) *X-rays and Polytomography*

The best plain films to show the nasopharynx are the A—P, the lateral and the submentovertical (Plate XI). Tomograms are useful to demonstrate bony destruction (Plate XII).

**PLATE XI**
**Submentovertical view showing invasion of both nasal fossae by tumour**

**PLATE XII**
**A—P tomograph demonstrating bony destruction by tumour**

(2) *Carotid angiography* is also of value in delineating the size and vascular quality of the tumour.

(3) *Biopsy* was carried out in three of our patients. However it is not always necessary and should only be undertaken with full facilities available to cope with excess bleeding.

**Treatment**

The number of therapeutic measures reported for dealing with the nasopharyngeal angiofibroma is testimony to the failure of most to produce the desired result. To quote Furstenberg and Boles (1963) 'those studies in research which have been enthusiastically announced have failed, with but few exceptions, to live beyond that feeble period which we term "the preliminary report".'

There are basically three methods of treatment which can be combined.

(1) *Hormonal.* There would seem to be little doubt that elaboration of male sex hormone has a positive influence on the development of angiofibroma in the tissues of the nasopharynx, and the hormonal effects upon connective tissues are well recognised. However, the results with hormonal therapy are variable and it would appear that, although some success in obtaining regression of the tumour has been achieved in a number of patients, in the main failure to influence the lesion is much more common (Butler et al, 1967). Two of the patients in this series received stilboestrol 5 mg. t.d.s. for one month pre-operatively. In one some regression of the tumour was evident.

(2) *Irradiation.* Again the results of irradiation (external and interstitial) are mixed and variable. In Conley's opinion (1968) irradiation alone or in combination with surgery is not the treatment of choice because of the late pernicious effects of radiation. Sarcomatous changes have been reported following this therapy. However there would seem to be no doubt that it can reduce the vascularity of the tumour in certain cases, and is sometimes given pre-operatively for this effect. One of our patients received 3,500 rads before being submitted to surgery. The blood loss at operation amounted to 600 ml. It is doubtful if the cytotoxic drugs at present available are of any significant value in the management of this tumour. One patient received 307mg methotrexate by intra-arterial infusion pre-operatively with apparently little effect.

(3) *Surgery.* Most authors agree that, at the present time, the treatment of choice for nasopharyngeal angiofibroma is surgical. As serious problems in planning a total extirpative procedure arise because of the anatomical extensions and involvements of the tumour, no one standard operation has been developed.

There have been three main avenues of approach to the nasopharynx: through the nose, through the upper jaw or through the mouth. Since Dupuytren in 1830 described an incision from medial canthus to medial canthus around the nasofacial sulcus, elevating the whole nose, and Ollier in 1866 used bilateral incisions of the nasofacial sulci connected across the bridge of the nose and turned the whole nose downwards, no less than 55 approaches to the nasopharynx have been reported in the literature. This diversity is surely proof of the unsatisfactory nature of any one approach. Obviously any useful route to the nasopharynx must give adequate exposure for best results. The tumour must be excised under direct vision if all remnants are to be removed. Although there is perhaps no single surgical technique which will solve all the problems which may be encountered, we feel that the *transpalatal approach,* using a transverse incision, as described by Wilson (1950), gives adequate access to the area, and is superior to any previously described in the literature. This was the method used in our four patients. No significant problems were encountered, and we were surprised at the ease with which the tumour was extirpated. Blood loss was not excessive in any of our patients. In one the *external carotid artery was ligated,* although some surgeons believe this to be of little value, as the vessels of the tumour communicate primarily with the venous system of the surrounding tissue (Butler et al 1967, Conley et al 1968). *Hypotensive anaesthesia* was used in three patients, *hypothermia* in one. There were no particular post-operative complications encountered in any of the four patients. A follow up from 10 years to 11 months has shown no evidence of recurrence of growth, and usually most recurrences will become manifest within two years.

Other approaches used and popular with some surgeons include *Moure's lateral rhinotomy* (1902), *Denker's extended operation* (1905), and the *Weber-Fergusson technique* (1866). A combined transpalatal, lateral rhinotomy, Caldwell-Luc method is sometimes used particularly if there are cheek and infratemporal fossa extensions. The line of approach in these techniques would appear to be directed too high to give a satisfactory exposure of the nasopharynx.

The more conservative operative procedures, such as the *transoral retropalatal approach* advocated by Furstenberg and Boles (1963), and the *combined intranasal-retropalatal technique* suggested by White (1955), do not seem to give as good results as the more radical transpalatal or lateral rhinotomy operations. The advent of *cryosurgical techniques* in the treatment of these tumours is promising and would seem likely to be of great value in specific instances, especially in the reduction of blood loss and in the management of small tumours and local recurrences (Work et al 1966).

**Conclusions**

Obviously no significant conclusions can be drawn from the treatment of four patients. However, at present in Bristol we favour surgical excision using the transpalatal approach, in the management of nasopharyngeal angiofibromas. Irradiation and endocrine therapy would seem to be of some value pre-operatively. Although *spontaneous regression* of these tumours may occur, it should not be considered as a method of management, nor practised in the presence of significant signs or symptoms.

**Acknowledgements**

I wish to express my thanks to Mr. D. Fairman and Mr. J. Freeman for access to their patients' records, and to Mr. W. Sweet of the Department of Medical Illustration, Southmead Hospital for the photomicrographs and photographic prints of the radiographs.

### REFERENCES

Bensch, H. (1878), 'Beitrage zur Berutheilung der Chirurgischen Behandlung der Nasenrachenopolypen'. E. Morgenstern, Breslau.

Brunner, H. (1942), Annals of Otology, Rhinology and Laryngology, **51**, 29.

Butler, R. M., Nahum, A. M., and Hanafee, W. (1967), Transactions of the American Academy of Ophthalmology and Otolaryngology, **71**, 92.

Conley, J., Healey, W. V., Blaugrund, S. M., and Perzin, K. H. (1968), Surgery, Gynecology and Obstetrics, **126**, 825.

Denker (1905), Archives of Laryngology, **17**, 221.

Dupuytren (1830), Journal de la Clinique. Quoted from Morrel Mackenzie's 'Diseases of the Throat and Nose', **2**, 522, 1880.

Furstenberg, A. C., and Boles, K. (1963), Transactions of the American Academy of Ophthalmology and Otology, **67**, 518.

Moure (1902), Revue de Laryngologie, p. 401.

Ollier (1866), Bulletin de la Societé Imperiale de Chirurgie de Paris, p. 263.

Osborn, D. A. (1959), Journal of Laryngology and Otology, **73**, 295.

Schiff, M. (1959), The Laryngoscope, **69**, 981.

Shaheen, H. B. (1930), Journal of Laryngology and Otology, **45**, 259.

Weber (1866), Die Krankheiten des Gesichts. Pitha and Billroth 'Chirurgie' Bd 3 I Abtheil A. Abschnitt III p. 206.

White, D. (1955), Archives of Otolaryngology, **61**, 326.

Willis, R. A. (1953), Pathology of Tumours, Butterworth, London.

Wilson, C. P. (1950), Proceedings of the Royal Society of Medicine, **44**, 353.

Work, W. P., Boles, R., and Nichols, R. D. (1966), Transactions of the American Academy of Ophthalmology and Otolaryngology, **70**, 922.

## Figures and Tables

**PLATE VII f1:**
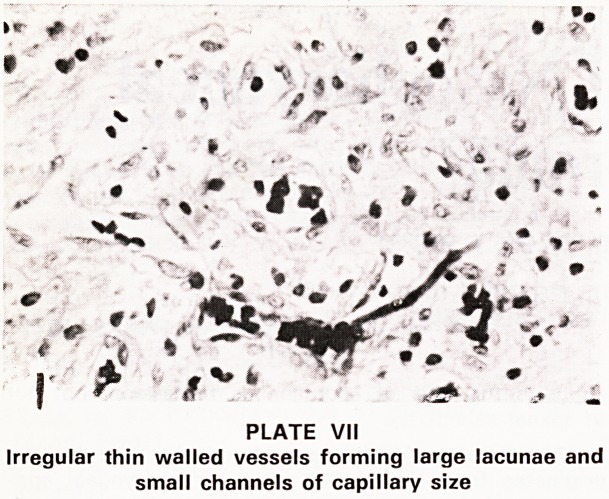


**PLATE VIII f2:**
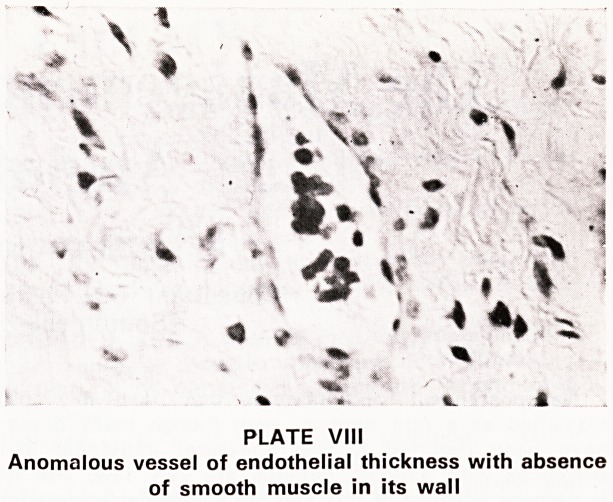


**PLATE IX f3:**
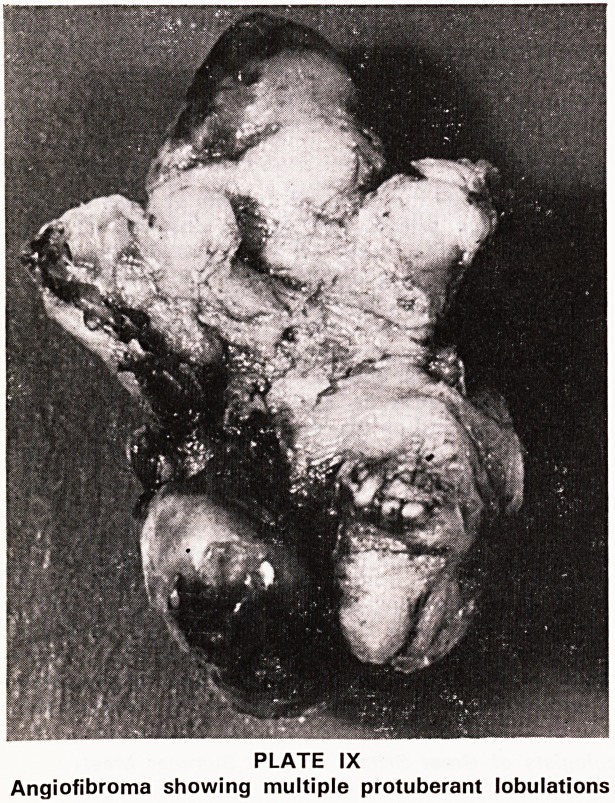


**PLATE X f4:**
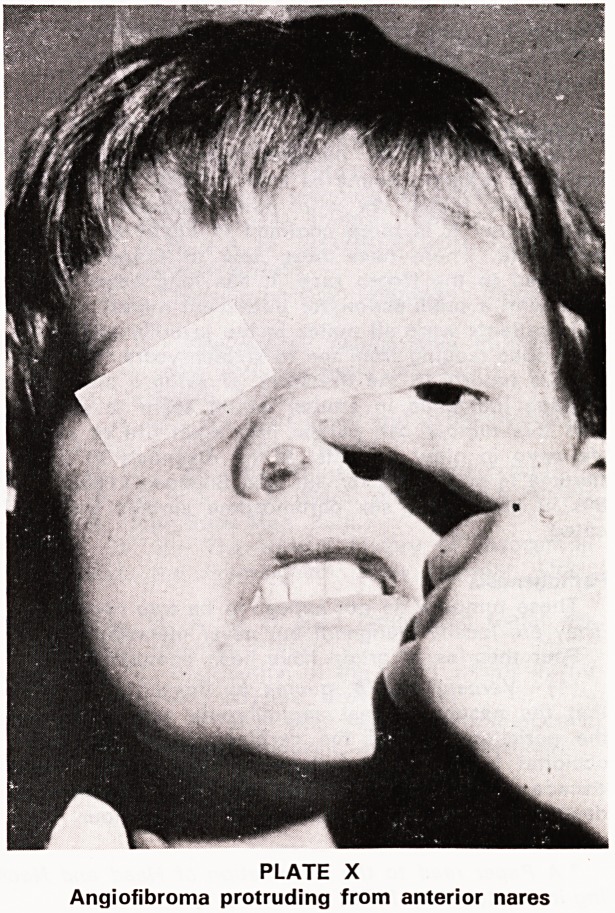


**PLATE XI f5:**
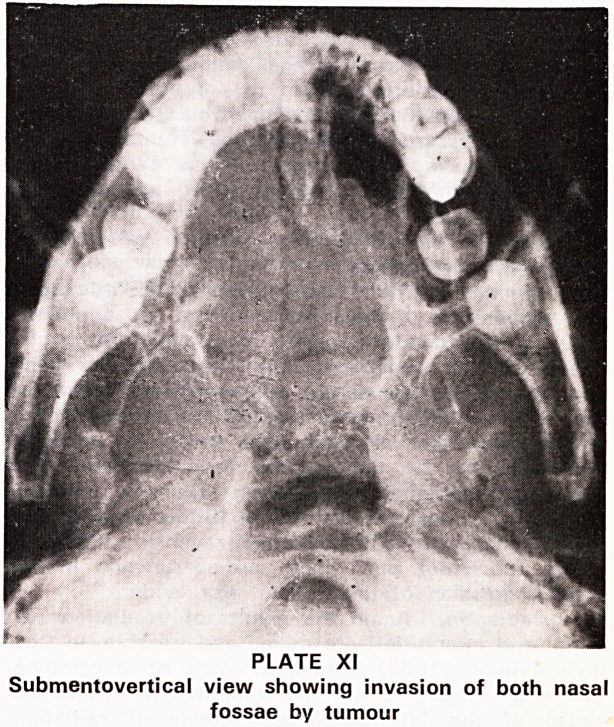


**PLATE XII f6:**